# Flight initiation distance and bird tolerance to humans in rural and urban habitats

**DOI:** 10.1098/rsos.240332

**Published:** 2024-10-09

**Authors:** Amrit Nepali, Hem Bahadur Katuwal, Sabin Kc, Sandeep Regmi, Hari Prasad Sharma

**Affiliations:** ^1^ Central Department of Zoology, Institute of Science and Technology, Tribhuvan University, Kirtipur, Kathmandu, Nepal; ^2^ Center for Integrative Conservation, Xishuangbanna Tropical Botanical Garden, Chinese Academy of Sciences, Mengla, Yunnan 666303, People’s Republic of China; ^3^ Nepal Zoological Society, Kirtipur, Kathmandu, Nepal

**Keywords:** feeding guild, flight initiation distance, flock size, rural, South Asia, urban

## Abstract

Urbanization induces homogenization and changes the behavioural patterns of various bird species, thereby facilitating coexistence and prompting adaptations to disturbances in urban environments. However, there is limited research on the influence of how urbanization affects bird tolerance towards humans, especially in developing sub-tropical regions such as Nepal, which is undergoing rapid unplanned urbanization. This study identified the flight initiation distance (FID) as a proxy for assessing bird tolerance. We focused on evaluating the human tolerance levels of 33 bird species using their FIDs in urban and rural habitats within Kathmandu Valley, a rapidly urbanizing city in South Asia. We found higher tolerance in urban birds than in their rural conspecifics, which varies mainly with dietary guild and season. The positive impact on FID was associated with time of the day and body size, while a negative association was observed with flock size, mean population density of humans and interaction between body size and elevation. Our study highlights the increased tolerance level of birds in urban areas, probably owing to habituation, and emphasizes the imperative need to investigate the potential adverse effect on urban bird population owing to this increased tolerance level.

## 1. Introduction

Rapid urbanization has resulted in habitat loss and fragmentation, with adverse effects on biodiversity [1]. Decreased green space with increasing anthropogenic activities has compelled wildlife, including bird species, to adapt to a changing environment that ranges from altered food sources to increased human presence [2]. Though some species alter their behaviour to cope with these habitat alterations [3], many species are unable to persist, particularly in response to urbanization [4]. Many bird species exhibit reduced tolerance towards direct human disturbance resulting in population declines [5] and threaten their persistence [6,7], which is evident from the several studies conducted across Europe and Australia [6,8–15].

Flight initiation distance (FID), the minimum distance at which birds flee when approached by humans or predators [6,10,16], is often regarded as a proxy for measuring bird tolerance towards humans. Further, it can also be a proxy for their reaction towards approaching disturbances and predators, as birds often view approaching humans as predators and in response they flee away [17]. This escaping behaviour of birds can be used to measure anti-predator strategies, monitor tolerance to disturbances and characterize human impacts on birds [6,18–20]. Despite being an anti-predatory response, the decision of birds to flee can reduce foraging efficiency and ultimately foraging success [21]. This is based on optimal escape theory [22]. Thus, understanding their FID helps to understand the relationships between birds and their adaptations in response to disturbances such as human presence or other potential predators. Further, understanding the tolerance levels of birds is particularly helpful for urban managers and policymakers to create suitable habitats in parks and also aids in the effective management of human disturbance [23].

While numerous studies have investigated the disparities in FIDs between rural and urban habitats [9,13,15,16,24–41], a comprehensive comparative analysis considering various life-history and eco-environmental factors across urban and rural habitats is noticeably absent. Though prevailing research indicates a greater tolerance (shorter FID) of species in urban settings [4,27,42–44], the magnitude of tolerance can vary based on different trait-based as well as ecological and environmental factors including but not limited to starting distance (SD), time of day, body size, flock size, sex, elevation, temperature, human population density, season, migratory status and dietary guild [6,16,24,40,45]. The influence of these variables varies across species and across urban and rural habitats [10,12,31,41,46,47]. However, studies of this nature are highly limited across Asian countries including Nepal.

We have limited understanding and notable gaps in current knowledge on bird tolerance in tropical Asian cities [44], although a few researchers have endeavoured to explore this aspect [26,37,39,40,48–50]. However, a conspicuous absence of studies prevails for South Asian sub-tropical cities like Kathmandu Valley of Nepal, where haphazard urbanization occurred without a comprehensive assessment of its impacts on biodiversity [51]. Birds in the valley are already exhibiting responses to the impacts of urbanization [52–54]; however, a notable gap exists in the current literature as no studies have been conducted to understand the tolerance of these birds to human presence across the urbanization gradients. In addition, most of the global studies conducted across the globe have analysed the tolerance of bird species across tropical habitats, and there is very little information available for bird species that share both urban and natural habitats across sub-tropical regions. The findings from this study will be valuable for enhancing the design of urban parks with site-specific management plans, as well as bird-focused protected areas, and mitigating the negative impact of bird–human interaction. Similarly, understanding the tolerance of birds with humans as a proxy of approaching danger can be considered in policymaking and decision-making processes related to environmental management and conservation action plans.

The primary goal of our study was to understand bird tolerance using FIDs in the urbanizing Kathmandu Valley of Nepal to provide baseline data for bird tolerance to humans in the sub-tropical city and fill gaps in the knowledge from the South Asian region. We specifically aimed (i) to compare FIDs of birds between urban and rural habitats based on sex, dietary guild, behaviour, season and time of the day, and (ii) to identify factors affecting the FID of birds in Kathmandu Valley, Nepal. As people feed many birds in urban habitats, especially in city parks and temples, and this practice is rarely seen in rural areas [16] and the risk of predation is higher in rural areas than in urban, we predicted greater tolerance for urban birds owing to habituation than rural ones. In accordance with the principles of dilution effect [55,56], we hypothesized longer FID with increase in flock size. Additionally, we assume that diurnal variations in FID will occur, with birds prioritizing foraging over vigilance during periods of high satiation, thus increasing FID in the morning [47,57]. Also, we anticipated that the avian population inhabiting regions with higher population density will display lower FID owing to habituation to human presence, especially beyond a certain threshold of human density. Furthermore, we postulate that migratory birds will exhibit slower adaptation to anthropogenic environments compared to resident species, resulting in decreased FID [48,58]. Also, we predicted that granivore species, which rely heavily on ground foraging in human-dominated areas, will demonstrate greater tolerance towards human presence compared to carnivorous species, which typically perch at heights away from human activity [39].

## 2. Material and methods

### 2.1. Study area

**Figure 1.**
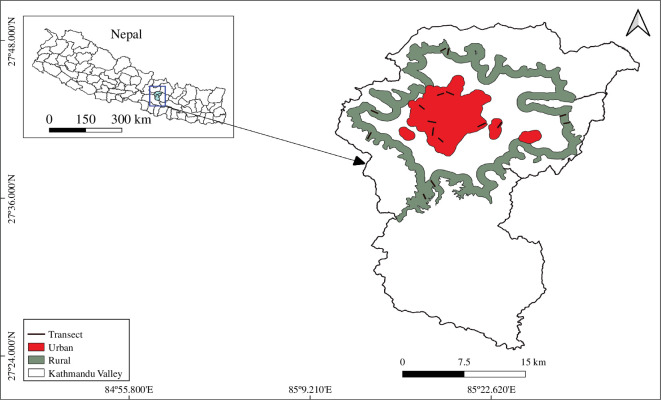
Study area with transect location in rural and urban habitats in Kathmandu Valley.

We conducted this study in Kathmandu Valley (27^o^24ʹ10ʺ to 27^o^48ʹ56ʺ N and 85^o^11*′*27ʺ to 85^o^34*′*15ʺ E), encompassing the administrative districts of Kathmandu, Bhaktapur, and Lalitpur of Nepal (figure 1). This area is in the mid-hills region with elevations from 1200 to 2760 m a.s.l. and temperatures from 0°C to 35°C. Kathmandu Valley provides a wide range of habitats for birds including forest, settlement, farmland, grassland, rivers and lakes [52,59,60]. It also holds a stopover site for winter migratory waterfowl and wetlands birds [60,61]. Owing to heterogeneity in habitats, the valley holds more than 500 species of birds [62]. The area is experiencing rapid urbanization with a significant increase in its urban population, reaching 2.5 million in 2018, which is more than one-fourth of the country’s total urban population [51] and is estimated to double by 2035 [63].

### 2.2. Research permission

We received the research permission from the Ministry of Forests and Environment, Department of Forests and Soil Conservation, Nepal (permission no.: 078/79-1325). Data for this research were collected without animal handling.

### 2.3. Research design and data collection

For this study, we divided the Kathmandu Valley into urban and rural areas based on land use characteristics following [52]. We buffered 1 km distance from the foothills of Kathmandu Valley towards the city area and defined it as rural areas, which primarily consist of extensive agricultural fields with only a few scattered buildings. On the other hand, urban areas were defined as areas inside the ring road, which are mostly urbanized with a continuous built-up environment separated by roads and city parks.

We assessed the bird tolerance using FIDs [16], following an established protocol [6]. We conducted observations for 32 days in winter (January‒February 2022) and summer (April‒May 2022) during favourable conditions (e.g. sunny, no precipitation, no winds) in the morning (07.00 to 11.00) and in the afternoon (14.00 to 17.00). We used eight urban and eight rural transects established by Katuwal *et al*. [52], each approximately 1 km of walking distance. For each transect, we followed existing roads and visited each transect twice a day, once each morning and afternoon, for 2 days, alternating between time periods in each season. We mainly focused on only diurnal birds that were involved in foraging or roosting behaviour and avoided juvenile birds to reduce potential bias owing to their shorter FID [5]. We focused our study on diurnal bird species owing to the relatively lower human disturbance at night in our study area. Measuring the FID using a range finder is challenging under low-light conditions, and nocturnal birds are not easily visible, which makes the data collection difficult. Our primary objective is to assess the tolerance capacity of diurnal birds, which are more frequently in proximity to humans, unlike nocturnal birds that are active only at night. Nocturnal birds have different behaviour and ecological requirements than diurnal birds, which require different research design as they are mainly affected by artificial lighting rather than human presence. We did not take photographs before estimating FID and used binoculars only for the observation as using a camera may evoke a longer FID in birds than without using camera [64]. We used a laser rangefinder with 4× zoom in order to estimate the FIDs. To estimate FID, we directly approached birds at a consistent pace of about 0.5 m s^−1^ in the direction where we first sighted the individual bird. We assumed the approaching human as a proxy of disturbance, predators, and threats to the birds. The SD was the initial distance from which the observer approached towards the bird. As previous studies [6,9] reported a significant relationship between FID and SD, we ensured that birds were approached from a minimum distance of 15 m (range: 15‒45 m). We recorded alert distance (AD) as the distance at which the bird first noticed the observer, and FID was recorded as the distance at which the bird fled on foot or took flight in response to the approach. We measured all distances of greater than 5 m using rangefinders and distances less than 5 m using a metre-length stick. During each observation, we also recorded the time of day, birds’ sex (if the species is dichromatic) and behaviour (foraging or roosting). When bird flocks were observed, we recorded data on the nearest bird. In cases where groups of birds representing multiple species were present, we approached the nearest individual, noted co-occurring species within a 5 m radius and recorded respective flock sizes [65]. To reduce the confounding effects of habituation, we did not approach birds within compounds of houses or temples as there might be another disturbance beside us that may trigger the flight in birds. We also discarded observations when another form of disturbance (e.g. potential predator) occurred simultaneously. As birds get alerted earlier to bright colour clothes [41], we wore neutral clothing to further reduce bias and did not approach birds carrying nesting material, nesting or attending young. We classified dietary guilds of bird species into insectivores, carnivores, granivores and omnivores following [66–68]. We extracted the information on body size and migratory status of recorded bird species from [67]. We extracted elevation from the digital elevation model map, and the data on human population density were extracted using 30 m resolution population density data from Humanitarian Data Exchange database [69] using QGIS [70]. The population density was extracted as an average within the 500 m radius from the centre of each survey transect.

### 2.4. Data analysis

We used *t*‐test and box plot visualization techniques to assess the difference in FIDs of bird species between rural and urban habitat constraints. For that, we used the residual FID values of each individual species derived from a phylogenetically controlled model and quantified the difference based on sex, dietary guild, season, activity type (foraging or roosting), and migratory status. The reason behind this was to account for phylogenetic bias that may occur owing to pseudoreplication. To model FIDs’ relationship with environmental and life-history factors of birds, we first did the phylogenetically controlled Bayesian Markov chain Monte Carlo generalized linear mixed modelling (MCMCGLMM) between FID and SD using package MCMCglmm [71] in the R statistical software [72]. For phylogenetic GLMM, we used phylogeny as a random effect for the dual purpose of mitigating the issue of non-independence among species and addressing the challenge of repeated sampling for the same species. It helps to incorporate the variation within species and minimize the sampling differences. For the phylogenetic tree, we downloaded a subset of pseudo-posterior distribution of trees from https://birdtree.org/ [73]. A total of 2000 trees using backbone phylogeny [74] were downloaded, and a single tree was generated using majority rule consensus phylogeny in Mesquite [75]. We used flat uninformative priors (*V* = 1, nu = 0.02) for both random terms and residual variance. The phylogenetic GLMM was performed using a total of 1 000 000 iterations with 1000 burn ins and a thinning value of 500. After phylogenetic MCMCGLMM, we performed generalized linear modelling using residuals of FID–SD regression as response variable and performed model selection using the dredge function in the MuMIn package [76]. We selected the best explaining model based on corrected Akaike’s information criterion (AICc). For model selection, we used factors like time of the day, body size, flock size, elevation, human population density, interaction between elevation and body size of birds, interaction between human population density and body size of birds, interaction between elevation and flock size as well as interaction between human population density and flock size. We omitted AD from our study as FID and AD were highly correlated (*r* = 0.92). To ensure a continuous variable for analysis, we converted the time of the day into radian time. Before analysis, the continuous variables were subjected to a correlation test with a threshold of *r* > |0.7|. Since elevation and temperature were highly correlated (|*r*| = −0.929), we omitted temperature from our analysis. Further, to account for the difference in measurements of the variables, we performed *z* standardization of the variables before analysis.

## 3 . Results

We collected 991 FIDs of 45 bird species; however, we included only those species with greater than four FIDs recorded, which resulted in 922 FIDs from 33 species (505 FIDs of 32 species from rural areas and 417 FIDs of 18 species from urban areas; electronic supplementary material, table S1). We observed focal individuals to exhibit an alert behaviour at distances of 4–37 m (10.769 ± 4.114), while FID ranged from 1 to 30 m (8.386 ± 4.625).

### 3.1. Bird tolerance in urban–rural habitats

**Figure 2.**
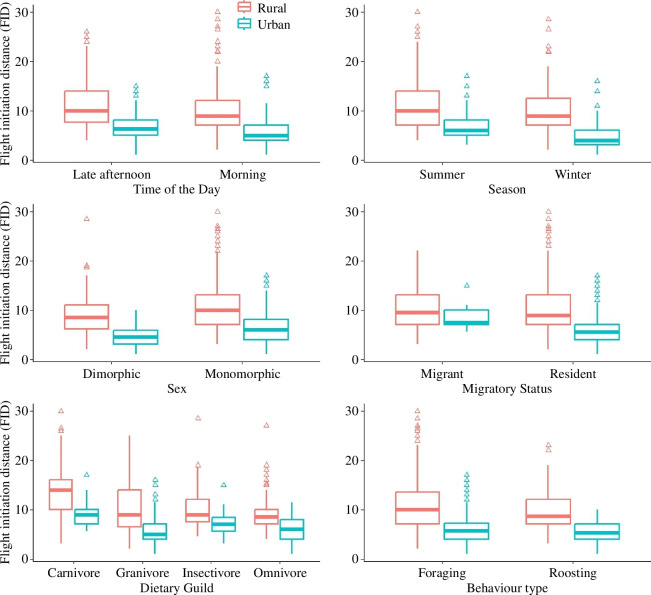
Comparison of flight initiation distance of birds across time of the day, season, sex, migratory status, dietary guild and behaviour type in Kathmandu Valley, Nepal.

We found a significantly higher bird tolerance in urban than in rural areas (FIDt = 19.043, *p *< 0.001), where the FID was 5.860 ± 2.684 m for urban and 10.357 ± 4.406 m for rural habitat. FID was also significantly longer in the late afternoon than in the morning (FIDt = −4.172, *p *< 0.001), during the summer compared to winter (FIDt = 3.917, *p *< 0.001), and for monomorphic species in comparison to dimorphic species (FIDt = −7.079, *p *< 0.001) and for migratory species compared to resident species (FIDt = −2.775, *p* = 0.007; figure 2). However, the FID did not differ from the activity pattern (FIDt = −0.413, *p* = 0.679). All these variables also showed significantly higher tolerance (shorter FIDs) in urban habitats than in rural (*p *< 0.001), except for migrant species (FIDt = 1.998, *p* = 0.051; see figure 2).

### 3.2. Factors affecting the bird tolerance

Our model selection showed that time, body size, flock size, elevation, mean human population density, interaction between body size and elevation and interaction between body size and human population density were the determining factors for FID of birds in the Kathmandu Valley (table 1). Among these factors, a significant impact was observed for time, body size, flock size, human population density and interaction between body size and elevation. FID of birds increased with an increase in radian time (0.05 ± 0.012) and body size (0.173 ± 0.013), implying a decreased tolerance with increased time and body size (table 2). However, there was a decline in FID with increasing flock size (−0.045 ± 0.014), human population density (−0.255 ± 0.017) and interaction between body size and elevation (−0.041 ± 0.016). These factors were observed to increase the tolerance level of birds in Kathmandu Valley, Nepal.

**Table 1.** Model selection table for flight initiation distance of bird species in Kathmandu Valley, Nepal. S.N.: Serial Number.

S.N.	model components	d.f.	AICc	delta	weight
1	time + body size + elevation + flock size + population density + body size × elevation + body size × population density	8	4856.203	0.000	0.222
2	time + body size + elevation + flock size + population density + body size × elevation + body size × population density	9	4856.920	0.717	0.155
3	time + body size + elevation + flock size + population density + body size × elevation + body size × population density + elevation × flock size	7	4857.787	1.584	0.101
4	time + body size + elevation + flock size + population density + body size × elevation	9	4858.211	2.008	0.081
5	time + body size + elevation + flock size + population density + body size × elevation + body size × population density + elevation × flock size + flock size × population density	10	4858.448	2.245	0.072
6	time + body size + elevation + flock size + population density + body size × elevation + elevation × flock size	8	4858.578	2.375	0.068
7	time + body size + elevation + flock size + population density	6	4858.790	2.587	0.061
8	time + body size + elevation + flock size + population density + body size × elevation + flock size × population density	8	4859.801	3.598	0.037
9	time + body size + elevation + flock size + population density + elevation × flock size	7	4859.862	3.659	0.036
10	time + body size + elevation + flock size + population density + body size × elevation + elevation × flock size + flock size × population density	9	4860.175	3.972	0.030

**Table 2.** Effects of variables on FID of birds in Kathmandu Valley, Nepal. (Values in bold represent significant association.)

parameters	estimate	s.e.	*z* value	*p*r(>|z|)
(intercept)	**1.941**	**0.013**	**149.750**	**<0.001**
time	**0.051**	**0.012**	**4.198**	**<0.001**
body size	**0.173**	**0.013**	**13.809**	**<0.001**
flock size	**−0.045**	**0.014**	**−3.215**	**0.001**
elevation	0.026	0.015	1.746	0.081
population density	**−0.255**	**0.017**	**−15.366**	**<0.001**
body size: elevation	**−0.041**	**0.016**	**−2.562**	**0.010**
body size: population density	−0.031	0.016	−1.941	0.052
flock size: elevation	0.013	0.009	1.384	0.166
flock size: population density	0.012	0.017	0.719	0.472

## 4. Discussion

Our study recorded a variation in tolerance level between rural and urban bird species as measured by their FID across various categories such as time of the day, season, sexual morphology migratory status, dietary guild and behaviour type at Kathmandu Valley, Nepal. The variation was in favour of our prediction, where we observed a reduced tolerance level with time and body size, whereas there was a positive association of bird tolerance with flock size, mean population density of humans as well as interaction of body size and elevation.

Our study identified that urban birds are less sensitive to human presence than their rural conspecifics, which might be owing to a low risk of predation from humans in urban areas than in rural [77]. Mostly people from rural areas are more involved in hunting birds than people from urban areas, which may increase the alertness. On the contrary, people in urban areas are often observed feeding birds around parks and temples, which might be perceived by birds as non-threatening to birds, thus enhancing acclimatization and reducing fear towards humans [16,38,78]. It could have important implications for their survival and reproductive success [35]. Further, urban bird species demonstrate a suite of adaptive traits, including smaller body size, diminished territorial behaviour, greater dispersal abilities, larger clutch sizes, longer lifespan and a more diversified dietary niche [27], which might collectively contribute to the capacity of urban birds to adapt in urban environments and thrive in human activities and habitat modification. The reduced territorial behaviour in urban birds can potentially facilitate coexistence with other birds in densely populated urban habitats; larger clutch size may serve as a compensatory mechanism for lower survival rates in urban habitats by producing more offspring [79], whereas longer lifespans may confer a capacity for urban birds to adapt to enduring changes within urban habitats over the long term [80]. Moreover, a higher bird abundance was observed in urban than in rural habitats [52], which helps to congregate their population and reduce tolerance [16].

Our study found birds were more tolerant in the morning than in the afternoon as we expected in both the comparative analysis as well as from modelling. This might relate to these species having higher resource and energy requirements during the morning [81]. At early hours, owing to long fasting throughout the night, bird species may primarily focus on accumulating energy through foraging, thus increasing tolerance towards humans in the morning, and low satiation levels in the day may increase the FID during the afternoon [47,57]. Generally, hunger can impair cognitive function, leading to decreased attention, memory and decision-making abilities [82]. Conversely, being well fed and satiated can improve cognitive performance, increasing alertness and focus [21]. Also, most of the predatory birds are more active during the daytime as they need warm air to take flight, which is readily available during the day [83]. So, this might make prey species increase their FID in the daytime to avoid detection and predation by these diurnal predators. Similar patterns of FID can be seen across multiple studies [40,47,48,84]. However, in contrast to our findings, Burger & Gochfeld [48] found shorter FID in the afternoon than in the morning.

We found longer FID of birds in summer than in winter, which might be owing to a potential correlation with increased access to food resources during the summer season [85]. So, birds are able to meet their daily energetic requirements easily in the summer season, and the balance in the trade-off between avoidance of starvation and predation shifts towards greater FIDs [86]. The summer season provides suitable climatic conditions that might decrease the caloric needs of birds, leading to an increase in FID [87]. In addition, the increased visibility in the summer season might help birds detect approaching predators and vice versa, and thus birds may reduce their tolerance level in order to reduce the risk of predation. Further, summer is also the breeding season for many birds [88], and birds respond differently during the breeding season to approaching predators because of parental investment in offspring and their direct fitness is associated with offspring survival [17]. Also, the high level of stress hormone (corticosterone) during the breeding season might affect their tolerance level towards humans [89,90].

Similarly, FID was longer in monomorphic birds than in dimorphic ones. This disparity owing to sexual dimorphism has also been illustrated by Møller *et al*. [91]. However, the higher tolerance (shorter FID) demonstrates the susceptibility of dimorphic species towards predation and helps the survival of the toughest individual, which can be seen to support the handicap hypothesis [92]. Indeed, individuals with more exaggerated secondary sexual characteristics are observed to experience lower predation rates, highlighting their superior quality [93]. However, our findings contrast with the study of Møller *et al*. [45], which suggests that birds with bright colours have a lower tolerance. We observed longer FID for migratory species in contrast to resident species, which might be owing to lesser familiarity with the environment for the migrants [48,58]. In addition, the high behavioural plasticity of residential birds may also make them able to tolerate human presence more than migrants [94].

Similarly, we observed the longest FID for carnivores and the shortest for granivores. This might be owing to the carnivores’ increased sensitivity to movement, which helps them to detect swiftly moving prey but also makes them more responsive to human presence [6]. Additionally, it could be that carnivore birds compete with humans for prey, which might make them less tolerant towards humans [16]. This indicated that carnivorous birds are more vulnerable to human-associated threats, which may have implications for their conservation. Granivores primarily feed on seeds, which are often concentrated in specific areas such as grassland or agricultural fields mostly dominated by humans, and they often encounter competition from conspecifics [39]. By maintaining a shorter FID, granivores may be able to gain competitive advantage by remaining close to the food source, reducing the energy costs associated with repeated take-offs and landing, maximizing their overall foraging gains. The omnivorous birds had longer FID than granivores but shorter than insectivores and carnivores, this might be owing to the interaction of these birds with humans. Omnivore birds have more interaction with humans as they also depend on food provided by humans, but insectivore birds have low interaction with humans as compared with granivores and omnivore birds and so are not adapted to human presence and exhibit longer FID with approaching humans to avoid predation risk by humans or other predators. These results are consistent with those of the previous studies [6,16,39,40], which suggest that dietary habit is an important factor in determining the sensitivity of birds to humans [10].

Further, FID of birds was positively influenced by time and body size. As previously mentioned, the longer FIDs observed later in the day probably reflect a satiation effect. The greater FIDs of larger birds than smaller birds might be owing to the increased detectability and reduced agility that makes them more vulnerable to predation and hence more likely to take flight [43,48,95]. Furthermore, larger birds with proportionally larger brains might exhibit enhanced cognitive capabilities, potentially enabling them to make more refined risk assessments before initiating flight [16]. By contrast, smaller birds tend to allocate more time to foraging owing to their relatively higher energy requirements [96]. This could explain their increased tolerance for risk before initiating flight. Interestingly, the negative association between FID and the interaction of body size and elevation suggests that the influence of body size on flight behaviour might be changed according to elevation.

The decrease in FID with an increase in flock size indicates birds perceive a high level of threats towards humans or predators with a decrease in flock size. It might be owing to a decrease in predation risk for individuals in the larger group, and predators are less likely to make a successful hunt on multiple targets owing to the dilution effect [55,56]. This finding agrees with a study in Europe that individuals in a group may benefit through risk dilution and so perceive a lower risk when in larger groups [97]. However, positive relationships have been reported between FID and flock size [14,98], which can be owing to the ‘many eyes effect’ hypothesis [99]. Similarly, the decrease in FID with increased population density suggests the habituation of bird species towards humans [16,28,40,100]. However, a recent study indicated that low human presence and disturbance in urban areas have increased FID rather than in rural counterparts [101]. This might be owing to the acclimatization of birds to human presence. From this, it can be perceived that the FID of birds might depend on the level of human disturbance or presence rather than the nature of the habitat itself. Thus, urban settings can also be used as a proxy of human intensity and predation risk rather than the nature of the habitat itself.

## 5. Conclusions

Our study highlights the effect of different life-history traits and eco-environmental variables on the FIDs of birds in Kathmandu Valley, Nepal. Urban birds with low FIDs were more tolerant of human presence than their rural counterparts. Birds have reduced tolerance with radian time and body size and have a positive association with flock size and human population density. These findings have important implications for bird conservation in urban habitats. Understanding the variations in FID and factors influencing bird tolerance towards humans can inform conservation strategies in urban environments. Creating suitable habitats and reducing disturbance levels in urban areas can contribute to the survival of both urban and rural bird species. Conservation efforts should consider the specific needs of different feeding guilds and their associated sensitivities to human activities. Furthermore, protecting natural habitats and promoting coexistence between humans and birds can help to maintain the biodiversity and ecosystem stability in urban landscapes. Overall, this study provides valuable insights into the dynamics of bird behaviour and human–bird interactions, aiding in the development of effective conservation measures for avian species in anthropogenic environments.

## Data Availability

Data are available in Dryad [102].
